# Genomic profiling reveals spatial intra-tumor heterogeneity in follicular lymphoma

**DOI:** 10.1038/s41375-018-0043-y

**Published:** 2018-02-08

**Authors:** Shamzah Araf, Jun Wang, Koorosh Korfi, Celine Pangault, Eleni Kotsiou, Ana Rio-Machin, Tahrima Rahim, James Heward, Andrew Clear, Sameena Iqbal, Jeff K. Davies, Peter Johnson, Maria Calaminici, Silvia Montoto, Rebecca Auer, Claude Chelala, John G. Gribben, Trevor A. Graham, Thierry Fest, Jude Fitzgibbon, Jessica Okosun

**Affiliations:** 1 Centre for Haemato-Oncology, Barts Cancer Institute, London, UK; 2 Centre for Genomic Health, Queen Mary University of London, London, UK; 3 Centre for Molecular Oncology, Barts Cancer Institute, London, UK; 4 UMR INSERM 1236, Université de Rennes, 1, EFS de Bretagne, CHU de Rennes, Rennes, France; 5 Cancer Sciences Unit, Cancer Research UK Centre, Southampton, UK; 6 Evolution and Cancer Laboratory, Barts Cancer Institute, London, UK

**Keywords:** Cancer genetics, Cancer genomics, B-cell lymphoma

Follicular lymphoma (FL) is an incurable B-cell malignancy characterized by advanced stage disease and a heterogeneous clinical course, with high-risk groups including those that transform to an aggressive lymphoma, or progress early (within 2 years) following treatment. Recent sequencing studies have established the diverse genomic landscape and the temporal clonal dynamics of FL [1–7]; however, our understanding of the degree of spatial or intra-tumor heterogeneity (ITH) that exists within an individual patient is limited. In contrast, multi-site profiling in solid organ malignancies has demonstrated profound ITH impacting mechanisms of drug resistance and compromising precision-medicine-based strategies to care [8]. In FL, the rise in trials adopting targeted therapies such as EZH2, PI3K, and BTK inhibitors reflects this paradigm shift in cancer care and with the development of biomarker-driven studies highlights the need to accurately define genomic alterations with clinical relevance. As most FL patients manifest disseminated tumor involvement, we sought to uncover the extent and clinical importance of spatial heterogeneity in FL by using a combination of whole-exome and targeted deep sequencing (Supplementary methods).

Our study cohort comprised nine patients (SP1–SP9) each with two spatially separated synchronous biopsies including two patients (SP3 and SP4) with spatial samples at two timepoints (FL and transformation), yielding a total of 22 tumor samples (Table S1). To improve the sensitivity for variant detection, fluorescence-activated cell sorting (FACS) was performed on cell suspensions where available (15 of 22 tumors) (Supplementary methods and Tables S2, S3). Exome sequencing of both the tumor and paired germline DNA was performed (median depth 131×) (Table S3) and we identified between 35 and 130 non-synonymous somatic variants (SNVs) per sample corresponding to 659 coding genes comprising missense (81%), indels (10%), nonsense (7%), and splice site (2%) changes (Tables S4, S5). We verified 195/198 (98%) SNVs using an orthogonal platform (Haloplex HS), with a high concordance of variant allele frequencies (VAFs) (*r* = 0.91) (Table S6). The tumor purity was predicted across samples using the mclust algorithm (Supplementary methods and Figure S1), demonstrating a mean purity of 92% in FACS-sorted samples and 66% in non-sorted samples.

Although the spatially separated tumors shared identical *BCL2*-*IGH* breakpoints, we observed variable degrees of ITH, with on average 82% (range 50–99%) of variants shared between sites. To quantify this heterogeneity, we calculated the Jaccard Similarity Coefficient (JSC) [9] for each patient, which represents the ratio of shared to total (shared and discordant) variants for two samples, with values closer to 1 representing greater similarity between samples. This demonstrated a range of JSCs with the highest JSC observed in SP3 (0.92) and the lowest in SP8 (JSC = 0.41) where a higher proportion of variants were confined to only one spatial biopsy (Fig. 1a, S2). Furthermore, the majority of our cases consisted of paired nodal/extra-nodal sites, and the extent of genetic heterogeneity may be more profound if additional nodal and extra-nodal sites of disease were profiled. The higher levels of genetic ITH in our study did not translate to a more adverse outcome nor was it associated with a specific clinical phenotype, although this can only be addressed with a larger series.

**Fig. 1:**
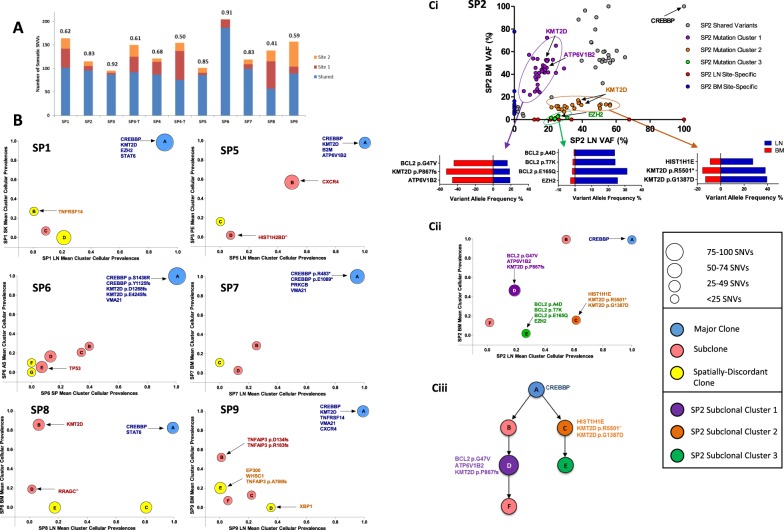
Patterns of intra-tumor heterogeneity in spatially separated tumors. **a** Proportion of shared and site-specific somatic SNVs in each case. The Jaccard Similarity Coefficient (JSC) is given above each bar. Site 1 is LN and site 2 BM with the following exceptions: SP1 site 2: skin (SK), SP4 site 1: LN1, site 2: LN2, SP4-T site 2: skin, SP5 site 2: pleural effusion (PE), SP6 site 1: ascites (AS), site 2: spleen (SP) (T: transformed). **b** Pairwise mean cluster cellular prevalence plots. Derived mutation clusters represent the mean cellular prevalence of all mutations within a cluster. Each cluster is denoted by a circle with the size of the circle equivalent to the number of mutations within the cluster. The letter in each circle relates to the specific cluster within the clonal phylogenies in Figure S3. Mutations in known FL-associated genes are highlighted to show their locations within clusters. ^^^Site-specific variant, although the mean cluster cellular prevalence is reported as marginally subclonal. **c** (i) Variant allele frequency (VAF) plot of all somatic mutations in case SP2. VAFs for selected mutations from three highlighted subclones in purple, orange, and green are shown in the horizontal bar graphs. **c** (ii) Mean cluster cellular prevalence plot and **c** (iii) clonal phylogeny of SP2 confirming the distinct subclones (purple, orange, green) seen in the VAF plot

To understand the clonal substructure of these spatially separated tumors, PyClone[16], a model-based clustering algorithm (Supplementary methods) was used to derive pairwise sub(clonal) clusters and reconstruct clonal phylogenies for each case (Fig. 1b, c and S3). This demonstrated tumors consisting of multiple subclones (mean 3, range 2–6), with the proportion of variants comprising the major clone (Fig. 1b) ranging from 6 to 68% (mean 40%). The non-linear distribution of subclones on the mean cluster cellular prevalence plots suggests differential subclonal dominance between spatial sites (Fig. 1b) and was best exemplified in SP2 where tumor cells from both compartments were FACS-purified. In this case, a variant cluster (Cluster 1) that included mutations in *ATP6V1B2* (p.R400Q), *BCL2* (p.G47V), and *KMT2D* (p.P867fs) were clonal in the bone marrow (BM) but subclonal in the lymph node, whereas the reverse was true for Cluster 2, consisting of mutations in *KMT2D* (p.G1387D and p.R5501*). We could also resolve a third cluster, including an *EZH2* mutation (p.Y646S), with corrected VAFs ranging from 21 to 31% in the lymh node (LN) and 0.6–2.6% in the BM (Fig. 1c).

Strikingly, in cases SP3 and SP4, where spatially separated biopsies were profiled at two timepoints (at FL and transformation), the spatial biopsies displayed strong genetic concordance pre-transformation; however, the degree of spatial heterogeneity markedly increased at transformation, with the JSC reducing from 0.92 to 0.61 and 0.68 to 0.50 in SP3 and SP4, respectively. Patient SP3 was treated with chemo-immunotherapy at diagnosis and relapsed 3 years later with transformed disease. Here, all four biopsies (spatial and temporal) shared mutations in *ARID1A*, *CREBBP*, *KMT2D*, 1p36 loss, and 17p gain (Fig. 2a, S4, and Table S7). There was evidence of devolution of specific genetic alterations at progression, with previously identified mutations in *ATP6V1B2* and *TNFRSF14* not observed, indicating that the transformed biopsies expanded from an ancestral population rather than directly from the dominant diagnostic clone. At transformation, shared temporal changes included acquisition of *REL* amplification, an *EZH2* mutation, and clonal expansion of a *CD79A* mutation that was present as a rare subclone at diagnosis. Spatial heterogeneity at transformation was illustrated by specific alterations in the transformed LN (tLN) including 6p copy neutral loss-of-heterozygosity (cnLOH) (encompassing the region encoding HLA genes) and mutations in *TNFAIP3*, *PRKCB* (p.R22H), and *DDX3×*. Following the same pattern as SP3, SP4 exhibited a core set of ubiquitous mutations in all biopsies (*CREBBP*, *EP300*, *KMT2D*, and *TNFRSF14*) with temporal loss of subclonal mutations in *PIK3CD* and *RRAGC*. There was a clear increase in ITH at transformation with both site-specific CNAs (Figure S4 and Table S7) and mutations in *EBF1*, *S1PR2*, *CCND3* (tLN), and *SPIB* (transformed skin (tSK)) (Fig. 2b). Interestingly, targeted sequencing of 13 selected variants in the circulating tumor DNA (ctDNA) sample at transformation detected mutations that were clonal and shared between the spatial biopsies (*CREBBP*, *KMT2D*, *EP300*, and *TNFRSF14*), but failed to recover all the site-specific variants in the tLN (e.g., *EBF1* and *S1PR2* corrected VAFs: 21.7% and 38.7%, respectively), indicating that different tumor subpopulations dynamically circulate in the plasma and that ctDNA may not invariably capture the entire genetic spectrum, and warrants further exploration (Figure S5).

**Fig. 2:**
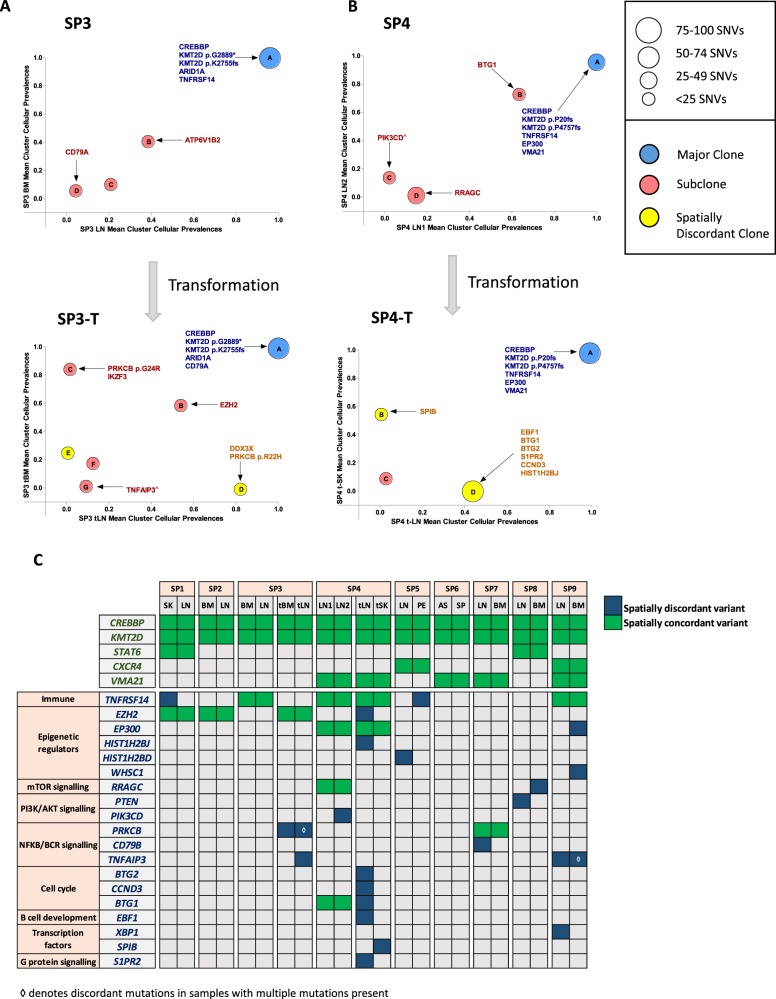
: Spatial heterogeneity at transformation and in genes with putative biological, prognostic, or therapeutic relevance. **a** Mean cluster cellular prevalence plot for SP3 at diagnosis (top) and transformation (bottom) to DLBCL. **b** Mean cluster cellular prevalence plot for SP4 at FL (top) and transformation (bottom) to DLBCL. ^^^Site-specific variant, although the mean cluster cellular prevalence is reported as marginally subclonal. **c** Heatmap demonstrating degree of spatial heterogeneity (mutations and copy number changes) in driver genes. At the top, alterations such as those in *CREBBP* and *KMT2D* are found in all cases. Gene names listed in green always had spatially concordant variants, while genes listed in blue demonstrate at least one instance of spatial discordance

To determine the clinical relevance of this spatial heterogeneity, we focused on known recurrently altered genes with putative biological, prognostic, or therapeutic relevance in FL (Fig. 2c). Notably, *CREBBP* was mutated in all nine patients, accompanied by cnLOH (seven cases) and was clonally maintained throughout spatially separated biopsies. This is in keeping with previous reports [2] and reaffirms *CREBBP* mutations as early events in the pathogenesis of FL. *KMT2D* was also affected by mutations or cnLOH in all cases, with a tendency for patients to possess multiple mutations with variations in clonality and evidence of genetic convergence with distinct mutations across spatial sites (Fig. 2c). In addition, *CXCR4* (SP5, SP9), *STAT6* (SP1, SP8), and *VMA21* (SP4, SP6, SP7, SP9) mutations were always spatially concordant. Aside from these genes, all others demonstrated spatial discordance in at least one case, with notable examples, including, site-specific mutations in *TNFAIP3* (SP3 and SP9), *TNFRSF14* (SP1), *PIK3CD* (SP4), *EP300* (SP9), *XBP1* (SP9), and copy number loss of *PTEN* (SP8) (Fig. 2c and S6). Of note, most discordant mutations were detected at a subclonal level (mean corrected VAF 27%; range 3.4–89%). We verified the site-specific and temporal-specific nature of these driver mutations identified from our exome data by performing ultra-deep sequencing of 25 selected variants (mean coverage 8,000×; Table S8 and Figure S7). All variants were confirmed to be truly spatially discordant at VAF sensitivities approaching 0.4%, apart from *CBX8* (SP5) confirming their bona fide site-specific nature.

Importantly, even accounting for the rarity of spatial sampling, reflecting the seldom nature spatially involved tumors are procured in routine clinical practice, the subclonal diversity and spatial heterogeneity observed in our case series has potential clinically relevant ramifications for the development of precision-based strategies, particularly in the context of emergent prognostic and predictive biomarkers. This is illustrated by examples of spatially discordant mutations in genes such as *EZH2* and *EP300* that are integral to the m7-FLIPI prognostic scoring model [10]. Furthermore, the heterogeneity of actionable driver events between sites may mean patients are precluded from adopting the relevant targeted therapy due to failure in the detection of the corresponding predictive biomarker in the solitary tumor biopsy profiled. A potentially attractive treatment paradigm is one whereby we specifically target highly recurrent and truncal gene mutations, such as *CREBBP* and *KMT2D*, particularly given their role in FL pathogenesis [11–14], as they may indeed prove to be the Achilles’ heel of these tumors.

In summary, this proof-of-principle study answers an important clinical question that a sole biopsy inadequately captures a patient’s genetic heterogeneity and prompts us to consider integrating multimodal genomic strategies (multiregion, ctDNA, and temporal profiling) into prospective clinical trials, as is currently being performed in the TRACERx study in lung cancer [15], especially as we begin to consider current and future actionable biomarkers.

## Electronic supplementary material

Supplemental Material

Supplementary Tables
