# Supplementary material for: Genomic profiling reveals spatial intra-tumor heterogeneity in follicular lymphoma
Source: Leukemia. 2018 Feb 8;32(5):1261–5. doi: 10.1038/s41375-018-0043-y (PMC5940637; doi:10.1038/s41375-018-0043-y)

**Supplementary Information**

# Genomic Profiling Reveals Spatial Intra-tumor Heterogeneity in Follicular Lymphoma

Shamzah Araf[1, 2*], Jun Wang[3*], Koorosh Korfi[1], Celine Pangault[4], Eleni Kotsiou[1], Ana Rio-Machin[1], Tahrima Rahim[1], James Heward[1], Andrew Clear[1], Sameena Iqbal[1], Jeff K. Davies[1], Peter Johnson[5], Maria Calaminici[1], Silvia Montoto[1], Rebecca Auer[1], Claude Chelala[3], John G. Gribben[1], Trevor Graham[6], Thierry Fest[4], Jude Fitzgibbon[1], Jessica Okosun[1]

[1]Centre for Haemato-Oncology, Barts Cancer Institute, London, United Kingdom,

[2]Centre for Genomic Health, Queen Mary University of London, London, United Kingdom

[3] Centre for Molecular Oncology, Barts Cancer Institute, London, United Kingdom,

[4]UMR INSERM 1236, Université de Rennes 1, EFS de Bretagne, CHU de Rennes, Rennes, France.

[5]Cancer Sciences Unit, Cancer Research UK Centre, Southampton, United Kingdom

[6]Evolution and Cancer Laboratory, Barts Cancer Institute, London, United Kingdom

*These authors contributed equally to the work

**Supplementary Methods**

**Patient selection and sample acquisition**

Twenty-two samples were collected from nine FL patients (SP1-9) who were selected on the basis of having available tumor biopsies from multiple disease sites and matched germline samples consisting of sorted T cells, peripheral blood or remission bone marrow (BM) (Table S1). Spatial samples from the same patient were all taken on average within 13 days of each other. The clonality between tumor biopsies was confirmed by *BCL2-IGH* breakpoint analysis in t(14:18) positive patients as previously described[1]. All biopsies were histologically reviewed to confirm diagnosis and tumor content before DNA extraction. The histological grade, where available, correlated between paired spatial samples except in the case of SP4 (Table S1). Written consent was obtained for the collection and use of specimens for research purposes with ethical approval obtained locally and within collaborating centers; (London Research Ethics Committee (LREC) of the East London and the City Health authority (10/H0704/65 and 06/Q0605/69) and Université de Rennes DC-2015-2565)

**Fluorescence activated cell sorting (FACS) and DNA extraction**

Frozen cell suspensions were available in 17/22 cases and used for tumor cell sorting to allow improved sensitivity for subclonal variant detection. Cells were isolated by FACS on an Aria II cell sorter (BD Biosciences) using the following cell surface markers based on the initial diagnostic immunophenotyping (Table S2); lymph node (LN), spleen (SP), pleural fluid (PE), ascites (AS): $CD20^+$ or $CD19^+$, $CD10^+$ and light chain restriction; BM: $CD20^+$ $CD10^+$ $CD38^+$ $CD44^{low}$ $CD34^-$ $CD138^-$ $IgD^-$ and light chain restriction. DNA was immediately extracted from cell suspensions using the DNeasy Blood & Tissue Kit (Qiagen) and from a formalin-fixed paraffin embedded (FFPE) sample (SP4-transformed skin (SP4-tSK)) using the Generead DNA FFPE Kit (Qiagen) according to the manufacturer's instructions. For samples with low DNA quantities, whole genome DNA amplification (WGA) was performed by the REPLI-g Mini Kit (Qiagen) prior to sequencing experiments (SP9-BM-whole exome sequencing, SP3-

tLN, SP3-tBM, SP4-LN1, SP5-LN, SP5-PE, SP9-BM-deep amplicon sequencing) according to the manufacturer's instructions.

**Whole exome sequencing (WES) and analysis**

Whole exome capture libraries were constructed from 200ng-3ug of tumor or germline DNA after shearing, end repair, phosphorylation and ligation to a barcoding sequencing adaptor, using the Human All Exon v5 SureSelect XT kit (Agilent Technologies). Enriched exome libraries were multiplexed and sequenced on the Illumina Hiseq 2500 platform to generate 100bp paired end reads. Sequencing metrics are provided in Table S3.

The processing and analysis of WES data were performed using our previously described pipeline [2, 3]. Briefly, sequencing reads were de-multiplexed and aligned to the reference genome hg19 using the Burrows-Wheeler Aligner (BWA) [4]. Local alignments and base quality scores were adjusted using the Genome Analysis Toolkit (GATK) version 2.5.2[5]. Somatic single nucleotide variants (SNVs) and insertion-deletions (indels) were identified using the Strelka pipeline[6]. For each patient, identified variants were further genotyped and verified across all tumors and matched normal samples together using the VarScan2 multi-sample calling method 'mpileup2cns'[7] based on high quality reads with mapping quality > 30 and minimum base quality of 20 at the targeted sites. The multi-sample calling step ensures that in cases where somatic variants were sufficiently identified in one of a spatial pair, the exact mutations were also included in the other sample of the pair when at least one high-quality supporting read was found. Additional criteria were used to call mutations in FFPE (SP4-transformed skin) and WGA (SP9-BM) samples and reduce the number of false positives, with the following variants excluded:

a) Variants with allelic fractions of less than 10% with the exception of variants that were identified in another biopsy from the same patient.

b) Site specific variants where the coverage/reads were less than 20 reads.

Annotation of somatic variants was obtained using the SNPnexus tool[8] and all non-synonymous somatic variants identified are listed in Table S5. WES data have been deposited into the European Genome-phenome Archive under the accession number EGAS00001002492.

**Somatic non-synonymous variant and coding indel validation**

Somatic non-synonymous variants were validated using a targeted FL gene panel (Table S9) designed for use with the Haloplex HS (Agilent Technologies) platform, according to the manufacturer's instructions. In brief, 50ng of genomic DNA was restriction enzyme digested before hybridization with the custom probe library for 16 hours and incorporation of molecular barcodes. Target DNA was captured and PCR amplified before multiplexing and sequencing on the Illumina Hiseq 2500 platform to generate 100bp paired end reads.

After de-multiplexing and de-barcoding, sequencing reads were processed using The Agilent Genomics NextGen Toolkit (AGeNT) (Agilent Technologies, http://www.genomics.agilent.com). Within the toolkit, the 'SurecallTrimmer' java application was first used to trim low quality bases from the ends, remove adaptor sequences and mask enzyme footprints. Filtered paired-end reads were then aligned to the reference genome hg19 using BOWTIE2[9]. After alignment, the AGeNT "LocatIt" program was used to process the molecular barcode (MBC) information and remove MBC duplicates from the alignment SAM files. The output of this step was coordinate-sorted alignment BAM files, with duplicates merged and output of only the consensus read pair sequence per MBC. The VarScan2 tool was used to examine the alignment pileup file to call variants against the reference genome based on high quality reads (same threshold as WES analysis) using a VAF cutoff of 3%. The strand bias filter was also applied. Identified variants were annotated using ANNOVAR[10] with nonsynonymous SNVs and coding indels further identified. Variants present in 1000 Genomes and NHLBI GO Exome Sequencing Project (ESP) with minor allele frequency (MAF) >1% were excluded. The remaining variants present in dbSNP138 and COSMIC v70[11] were then marked. Variants present in greater than three samples/patients were excluded unless they were known COSMIC cancer mutations. The filtered

nonsynonymous variants were then compared with those called by WES and any variants not identified were further inspected in the deep sequencing data using Integrative genomics viewer (IGV)[12] and included if manually visualized. Validated variants are listed in Table S6.

**Deep amplicon sequencing and analysis**

To obtain higher depth of coverage and accurately detect the presence or absence of spatially discordant mutations, deep sequencing of 25 selected genes were performed. Universal adapter sequences were tagged to the 5' and 3' ends of target specific primers. Genomic DNA (50-100ng) was amplified either in singleplex or 2-4 plex using the HotStar Taq Plus Kit (Qiagen) under limited cycling conditions. Amplified PCR products were subsequently pooled in equimolar ratios per sample and prepared for sequencing with the attachment of sample specific indexes and Illumina adaptor sequences. Indexed libraries were pooled and sequenced on the Illumina Miseq instrument using the v2 300 cycle conditions generating 150bp paired end reads. Whole genome amplified and FFPE samples were screened in duplicate and normal tonsil DNA controls were included for each primer pool.

Sequencing reads were aligned to the reference genome hg19 using BOWTIE2. SAM to BAM conversion was performed using Picard, followed by Indel realignment and base quality recalibration with GATK. VarScan2 was used for variant calling, selecting reads with a mapping quality > 20 and base quality score > 15. A minimum coverage of 100 reads and 10 variant supporting reads were required for the variant sites, with a minimum VAF threshold of 0.1% (~8,000x coverage of high quality reads was achieved). This threshold enabled sufficient detection of subclonal mutations within the spatial samples, but also allowed measurement of the background sequencing artefact or noise rate across each amplicon, which was estimated to be 0.15% - 0.40% across amplicons. The procedure to accurately detect low frequency mutations across samples has been described previously[13]. Briefly, the number of reads supporting the reference allele and that supporting the alternative allele were compared between the candidate sample and control DNA samples, as well as against the relevant

spatial samples. The intra-amplicon comparison with nearby variants was also performed using Fisher's exact test, allowing two-tailed $p < 0.05$.

**Copy number analysis**

Two independent approaches, (ASCAT[14] and VarScan2 'copynumber' and 'copyCaller' modules followed by the DNAcopy R Bioconductor package version 1.50.1), were employed to analyze tumor and matched normal pairs and identify copy number aberrations (CNA) and copy neutral loss-of-heterozygosity (cnLOH) as previously described[3,15]. In brief, the following criteria were used to discriminate copy number amplifications and losses:

a) ASCAT: amplification= (predicted total copy number - predicted ploidy) > 0.6; loss= (predicted total copy number - predicted ploidy) <- 0.6.
b) DNAcopy circular binary segmentation (CBS) analysis: amplification= logR ratio > 0.15; loss= logR < -0.15.

Consensus calls based on the output of these two methods were subsequently generated. In addition we visually inspected all copy number aberration events using the logR and B-allele frequency (BAF) plots, and further included copy number gain and loss events when they were clearly supported by one method, but just below the cutoff threshold in the other. Copy number analysis was not performed for sample SP9-BM as WES was performed on whole genome amplified DNA, affecting the quality and reliability of the copy number analysis described.

**Tumor content estimation**

We estimated the tumor content for each sample based on the variant allele frequencies (VAFs) of WES variants. Density estimation was performed via Gaussian finite mixture modelling using 'mclust' R package[16], and the number of VAF clusters was determined based on the Bayesian Information Criterion (BIC). The first major cluster with VAFs at the density peak just below 50% was regarded as the clonal cluster of heterozygous mutations (supplementary Figure 1). The tumor content was

estimated as twice the VAF value at the clonal peak (Table S3). To validate the mclust derived tumor content estimation we used SciClone[17], a variational Bayesian beta mixture modelling method, and obtained a high degree of concordance between both methods (r = 0.93) (Table S3).

**Clonality analysis and phylogenies**

Paired spatial samples were used to infer the mutational cellular prevalence of identified somatic variants (shared and unique) using PyClone (v0.13.0)[18]. Read counts for the reference and variant alleles, the total copy numbers for identified somatic variants and the estimated tumor content were input into the PyClone analysis using the "run_analysis_pipeline" command. Mutations with similar mutational cellular prevalences were further clustered by PyClone in two-dimensions and mean cluster cellular prevalences were calculated for both samples in a spatial pair. We filtered out clusters with ≤2 mutations which were always synonymous variants with no predicted functional impact. The "total copy number" setting was applied in PyClone, resulting in the cluster including *CREBBP* mutations affected by copy neutral LOH merging with the dominant clonal cluster comprising heterozygous mutations. Clonal phylogenies were constructed for each tumor pair based on the PyClone clustering patterns and cluster means (Supplementary Figure 3) and were compared with phylogenetic trees generated using PhyloWGS[19].

**Circulating tumor DNA (ctDNA) targeted re-sequencing**

Blood was collected into BD Vacutainer K2E (EDTA) tubes (Becton-Dickinson). Plasma was extracted by centrifugation at 1,600 × *g* for 10 minutes at room temperature and further purified by centrifugation at 4,500 × *g* for 10 minutes at room temperature, and stored at −80°C within 4 hours of collection. ctDNA was extracted using the QIAamp Circulating Nucleic Acid Kit (Qiagen) following the manufacturer's instructions, quantified by the TaqMan RNAseP Assay (Thermo Fisher Scientific) and stored at −20°C. Sequencing libraries were generated from 10 ng ctDNA, using the Accel-NGS 2S

Hyb DNA Library Kit with the 2S SureSelectXT MID Compatibility Module (Swift Biosciences) following the manufacturer's instructions with the following modifications. Library amplification and indexing was carried out with KAPA HiFi HotStart PCR Kit (Kapa Biosystems) and NEBNext Index Primers for Illumina (New England Biolabs). PCR-amplified libraries were quantified by Qubit 2.0 Fluorometer (Thermo Fisher Scientific) and 93.75ng was used in the hybrid capture reaction using the SureSelectXT2 Reagent Kit (Agilent Technologies) and a custom FL gene panel (Table S7), following the manufacturer's instructions. Captured libraries were amplified using the KAPA HiFi HotStart PCR Kit and PE1 (5′-AATGATACGGCGACCACCGAGATCT-3′), PE2 (5′-CAAGCAGAAGACGGCATACGAGAT-3′) primers. Libraries were paired-end sequenced generating 2 x 75bp reads on an Illumina NextSeq 500 using a High Output kit (Illumina). The pipeline used to identify variants from the ultra-deep sequencing data was also applied to the ctDNA sequencing readout.

**References**


1. Ladetto M, Sametti S, Donovan JW, Ferrero D, Astolfi M, Mitterer M*, et al.* A validated real-time quantitative PCR approach shows a correlation between tumor burden and successful ex vivo purging in follicular lymphoma patients. *Exp Hematol* 2001 Feb; **29**(2)**:** 183-193.
2. Okosun J, Bödör C, Wang J, Araf S, Yang CY, Pan C*, et al.* Integrated genomic analysis identifies recurrent mutations and evolution patterns driving the initiation and progression of follicular lymphoma. *Nat Genet* 2014 Feb; **46**(2)**:** 176-181.
3. Okosun J, Wolfson RL, Wang J, Araf S, Wilkins L, Castellano BM*, et al.* Recurrent mTORC1-activating RRAGC mutations in follicular lymphoma. *Nat Genet* 2016 Feb; **48**(2)**:** 183-188.
4. Li H, Durbin R. Fast and accurate short read alignment with Burrows-Wheeler transform. *Bioinformatics* 2009 Jul; **25**(14)**:** 1754-1760.
5. DePristo MA, Banks E, Poplin R, Garimella KV, Maguire JR, Hartl C*, et al.* A framework for variation discovery and genotyping using next-generation DNA sequencing data. *Nat Genet* 2011 May; **43**(5)**:** 491-498.
6. Saunders CT, Wong WS, Swamy S, Becq J, Murray LJ, Cheetham RK. Strelka: accurate somatic small-variant calling from sequenced tumor-normal sample pairs. *Bioinformatics* 2012 Jul; **28**(14)**:** 1811-1817.
7. Koboldt DC, Zhang Q, Larson DE, Shen D, McLellan MD, Lin L*, et al.* VarScan 2: somatic mutation and copy number alteration discovery in cancer by exome sequencing. *Genome Res* 2012 Mar; **22**(3)**:** 568-576.
8. Dayem Ullah AZ, Lemoine NR, Chelala C. SNPnexus: a web server for functional annotation of novel and publicly known genetic variants (2012 update). *Nucleic Acids Res* 2012 Jul; **40**(Web Server issue)**:** W65-70.
9. Langmead B, Salzberg SL. Fast gapped-read alignment with Bowtie 2. *Nat Methods* 2012 Mar; **9**(4)**:** 357-359.
10. Wang K, Li M, Hakonarson H. ANNOVAR: functional annotation of genetic variants from high-

throughput sequencing data. *Nucleic Acids Res* 2010 Sep; **38**(16)**:** e164.

11. Forbes SA, Beare D, Boutselakis H, Bamford S, Bindal N, Tate J*, et al.* COSMIC: somatic cancer genetics at high-resolution. *Nucleic Acids Res* 2017 Jan; **45**(D1)**:** D777-D783.
12. Robinson JT, Thorvaldsdóttir H, Winckler W, Guttman M, Lander ES, Getz G*, et al.* Integrative genomics viewer. *Nat Biotechnol* 2011 Jan; **29**(1)**:** 24-26.
13. Tawana K, Wang J, Renneville A, Bödör C, Hills R, Loveday C*, et al.* Disease evolution and outcomes in familial AML with germline CEBPA mutations. *Blood* 2015 Sep; **126**(10)**:** 1214-1223.
14. Van Loo P, Nilsen G, Nordgard SH, Vollan HK, Børresen-Dale AL, Kristensen VN*, et al.* Analyzing cancer samples with SNP arrays. *Methods Mol Biol* 2012; **802:** 57-72.
15. Cammareri P, Rose AM, Vincent DF, Wang J, Nagano A, Libertini S*, et al.* Inactivation of TGFβ receptors in stem cells drives cutaneous squamous cell carcinoma. *Nat Commun* 2016 Aug; **7:** 12493.
16. Fraley C, Raftery AE. Model-based clustering, discriminant analysis and density estimation. *J. Am. Stat. Assoc*. 2002; 97611–631
17. Miller CA, White BS, Dees ND, Griffith M, Welch JS, Griffith OL, et al. SciClone: inferring clonal architecture and tracking the spatial and temporal patterns of tumor evolution. PLoS Comput Biol 2014 Aug; 10(8): e1003665.
18. Roth A, Khattra J, Yap D, Wan A, Laks E, Biele J, et al. PyClone: statistical inference of clonal population structure in cancer. Nat Methods 2014 Apr; 11(4): 396-398.
19. Deshwar AG, Vembu S, Yung CK, Jang GH, Stein L, Morris Q. PhyloWGS: reconstructing subclonal composition and evolution from whole-genome sequencing of tumors. Genome Biol 2015 Feb; 16: 35.

**Supplementary Figures**

**Figure S1: Tumor content estimation and (sub)clonality classification for SP1-LN.** We computed tumor content for each sample based on WES variants. Density estimation was performed via Gaussian finite mixture modelling using ‘mclust’ R package, and the number of VAF clusters was determined based on the Bayesian Information Criterion (BIC). Cluster A with VAFs at the density peak just below 50% were regarded as clonal heterozygous mutations and tumor content was estimated as twice the VAF value at this clonal peak.

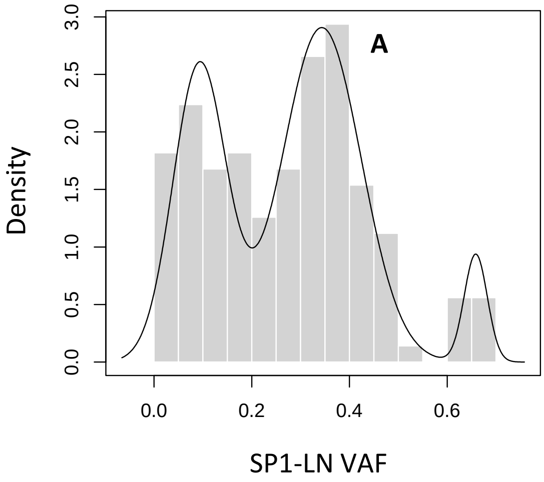

**Figure S2: Dot plots comparing the variant allele frequencies (VAFs) between spatial sites.** Each variant plot compares the VAFs of all synonymous variants between spatial sites with color-coded distributions. Site-specific variants are represented by either blue data points (y-axis) or red data points (x-axis). Shared variants are shown in grey. Mutations in known FL-associated genes are highlighted. SP2 is shown in Figure 1C.

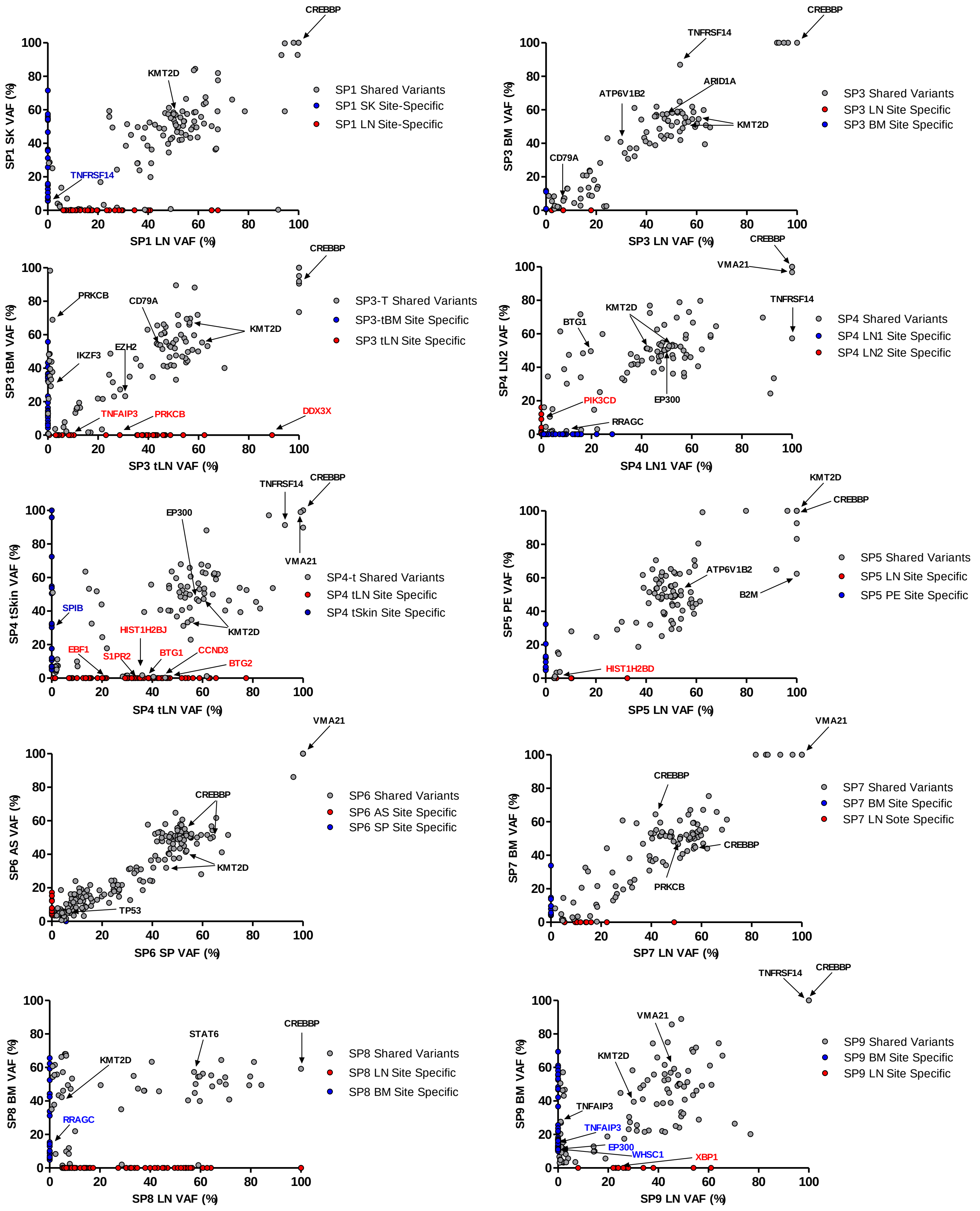

**Figure S3: Clonal phylogenies of spatially separated tumors.** Clonal phylogenies were constructed as described in the supplementary methods. Letters in each clone denote the same clone in Figures 1B, 1C, 2A, 2B. ^site specific variant, although the mean cluster cellular prevalence is reported as marginally subclonal. SP2 is shown in Figure 1C.

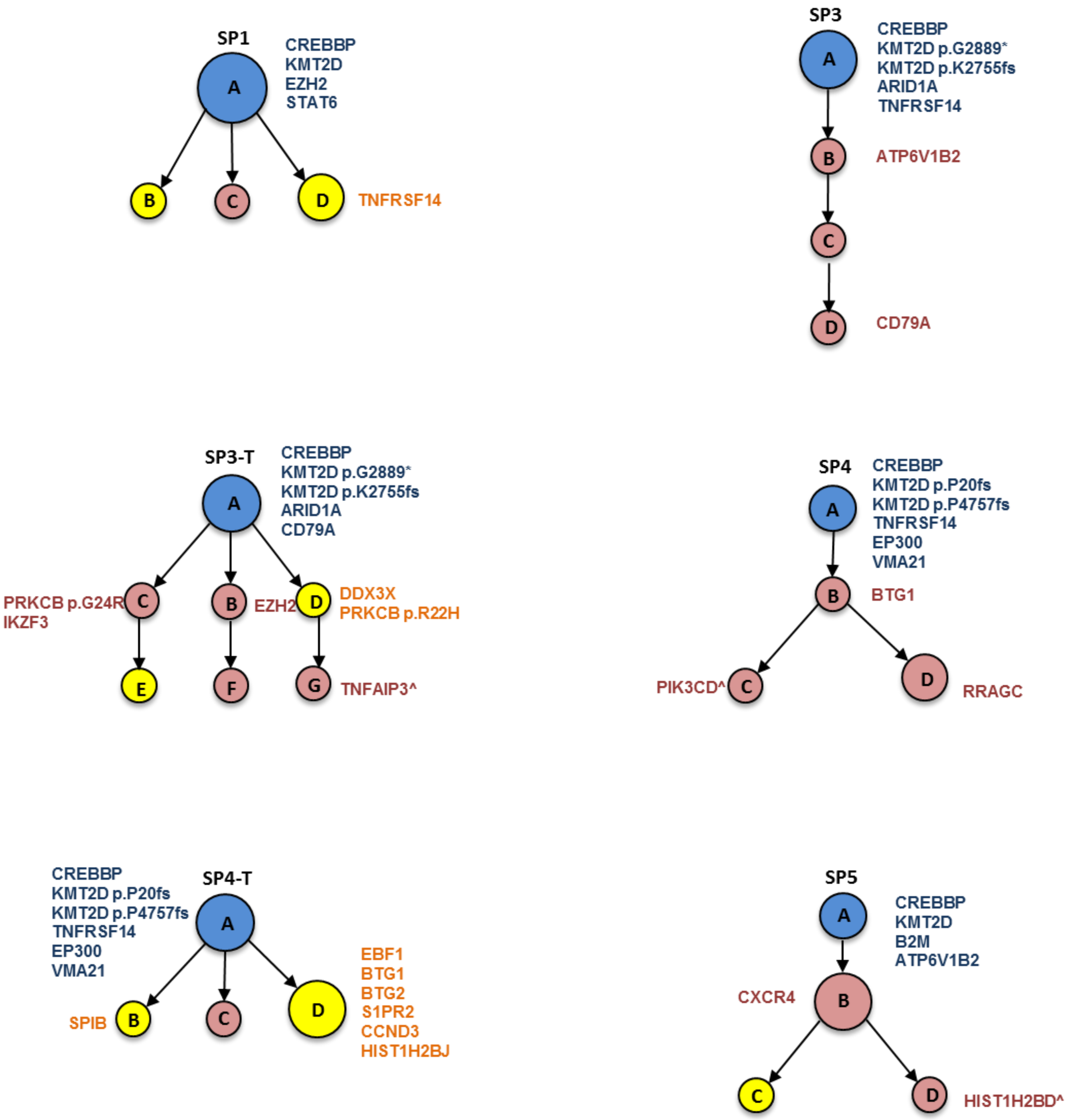

**Figure S3 continued**

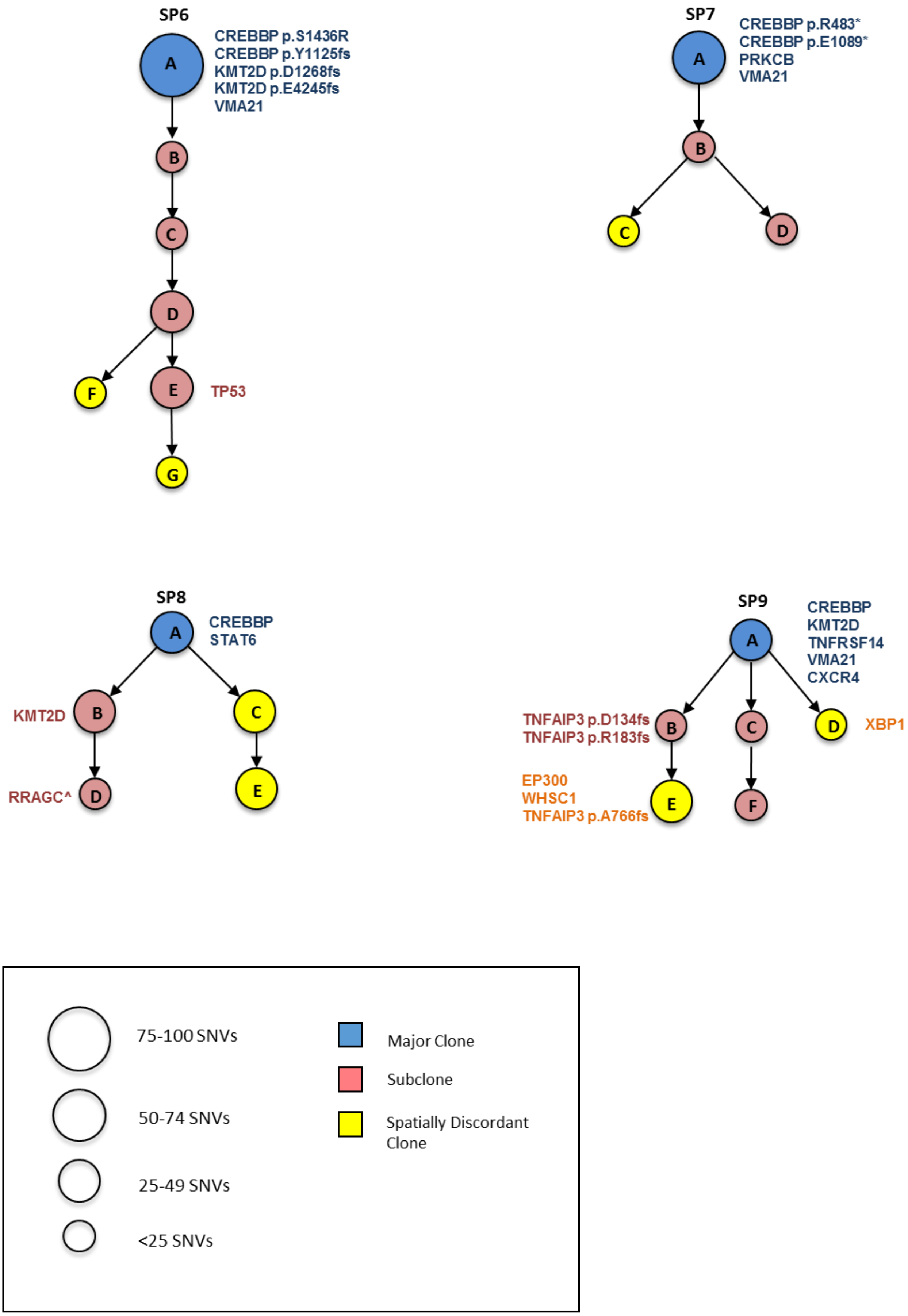

**Figure S4: Copy number changes in the 8 paired spatial tumors.** CNLOH (purple), amplifications (red) and deletions (blue) are shown for each paired spatial case, demonstrating regions of spatial discordance**.** Regions of interest are labeled with the candidate genes mapped from UCSC Genome Browser (GRCh37/hg19). Data for SP9 is not shown as whole genome amplified DNA from the BM was used for WES.

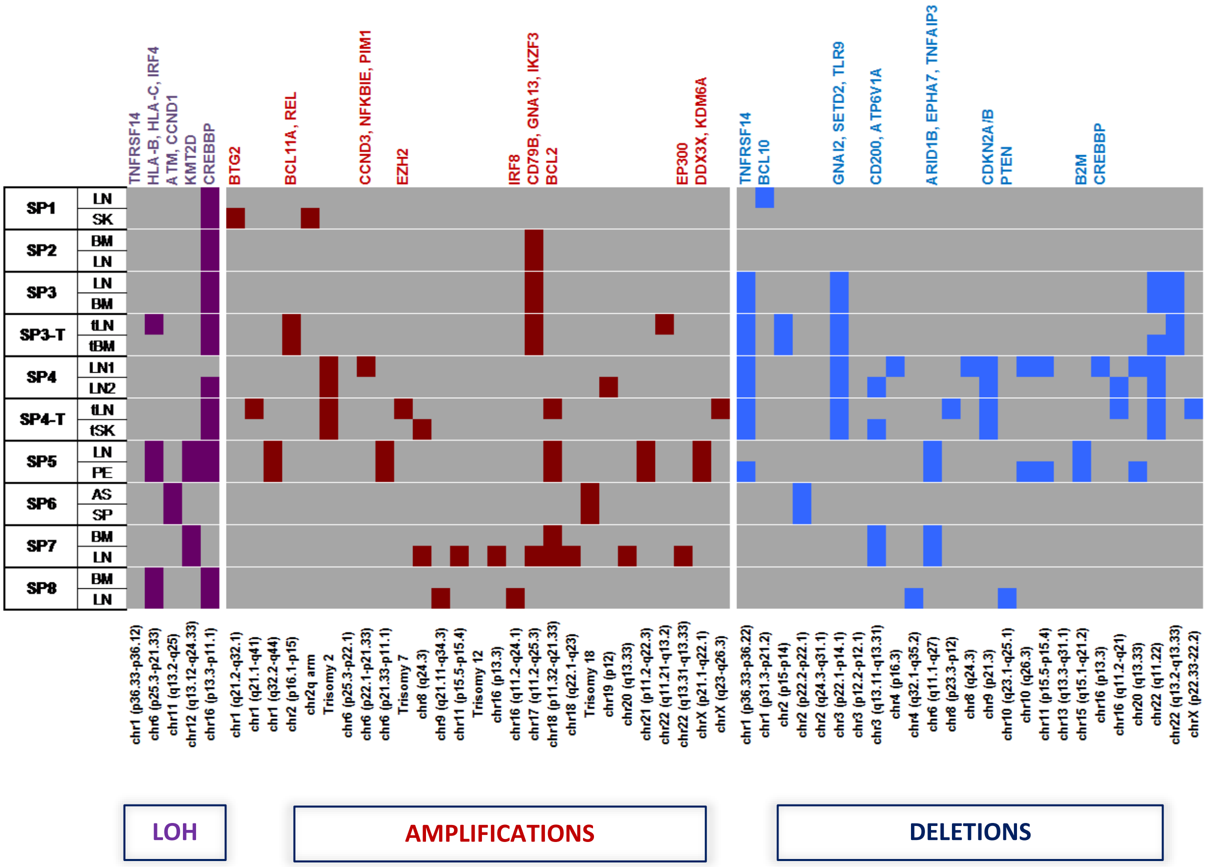

**Figure S5: Bar graph displaying results from targeted sequencing of ctDNA in SP4 at transformation.** Variants present in all biopsies (LN1, LN2, t-LN, t-Skin) are denoted by the grey bars. Variants spatially heterogeneous and in the transformed LN only are shown in red: *BTG1* was detected at a VAF of 15%, *BTG2, CCND3* and *HIST1H2BJ* were detected at low VAFs and *EBF1* and *S1PR2* were not detected. Variants present in the original FL and not in the transformed biopsies (*PIK3CD*, *RRAGC*) were not detected. [1]*KMT2D* p.P20fs, [2]*KMT2D* p.P4757fs.

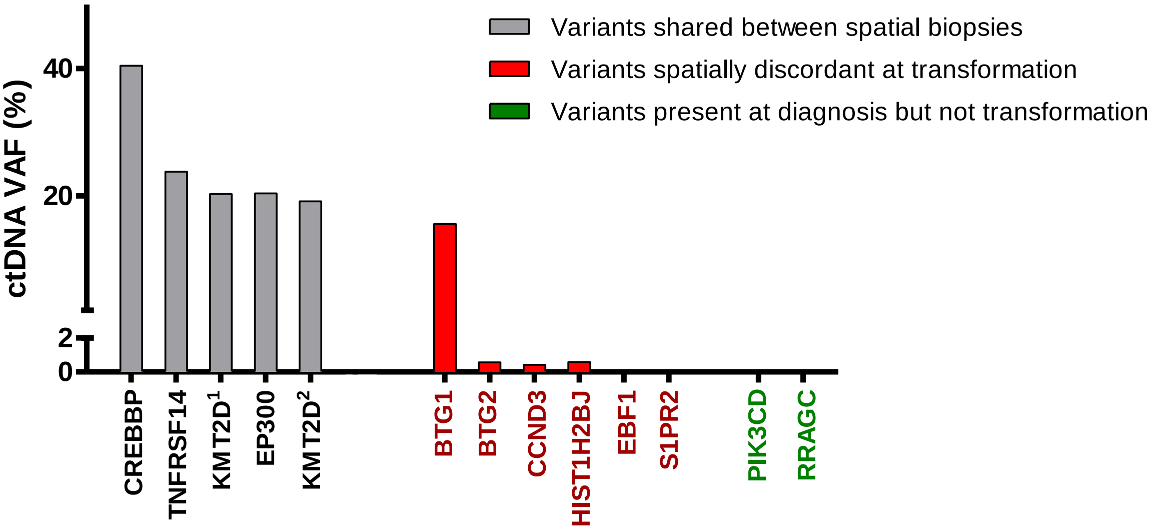

**Figure S6: Copy number alteration in SP8 demonstrating spatially discordant *PTEN* loss.** VarScan2 copy number and DNAcopy logR profile using WES data for SP8 LN and BM. Copy number loss of a region in chromosome 10 is observed in the LN but not the BM. The deleted segment is ~24Mb in length (segment coordinates: chr10:83,926,592 – 108,924,136) and encompassed the *PTEN* locus.

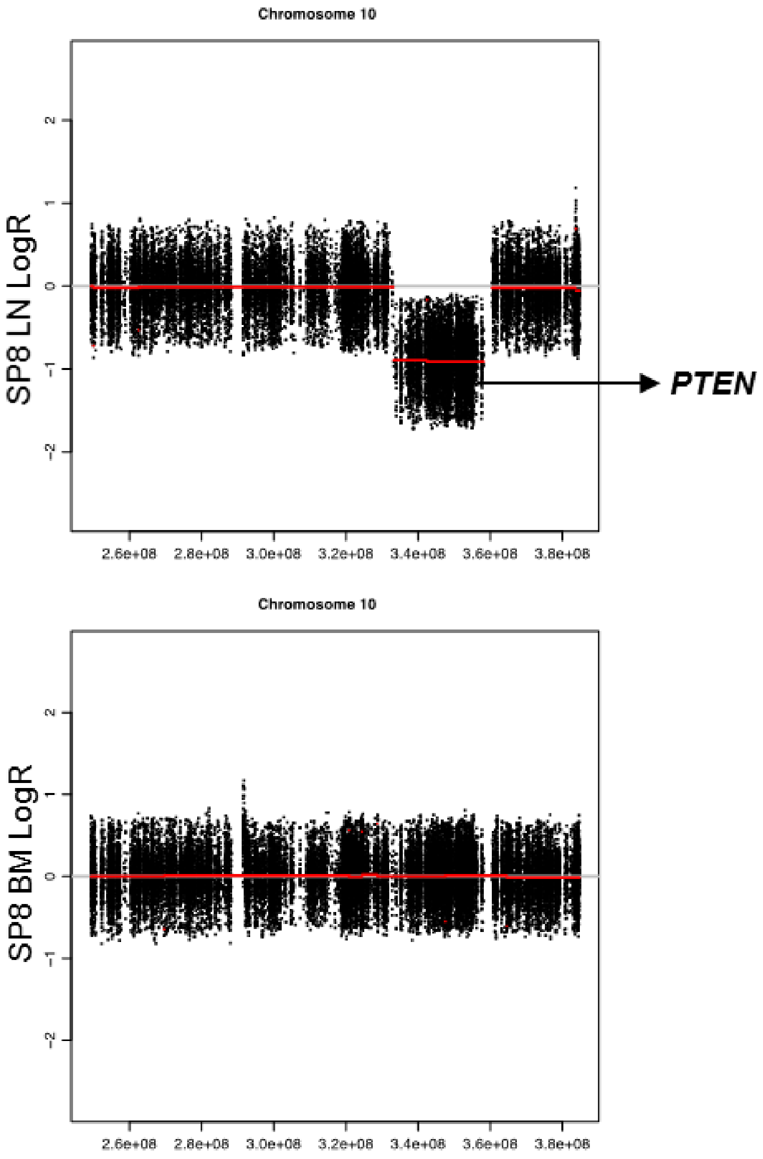

**Figure S7: Representative example of ultra-deep sequencing results covering the region of the variant *EP300* p.P2322fs in SP9.** No variant reads were detected in the LN (coverage 7998x) (top panel) but was identified in the BM at a VAF of 10% (bottom panel). This confirms WES data that demonstrated VAFs of 0% and 12.5% in the LN and BM respectively. The red dashed line denotes the upper 95% confidence interval of background sequencing noise

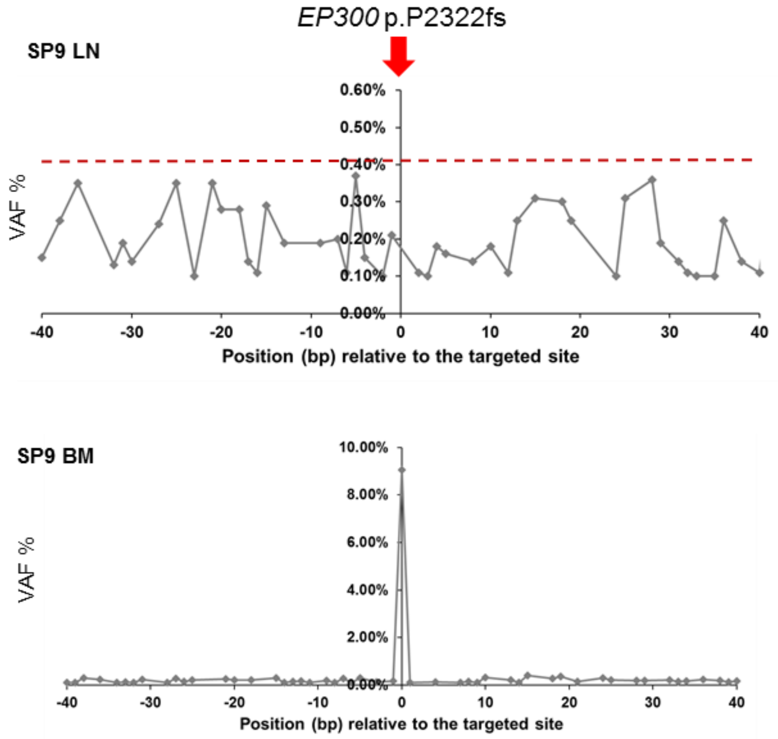

Supplement: Supplementary file 1 — Supplemental Material [file 41375_2018_43_MOESM1_ESM.pdf]
